# Supplementary material for: Analysing trajectories of a longitudinal exposure: A causal perspective on common methods in lifecourse research
Source: PLoS One. 2019 Dec 4;14(12):e0225217. doi: 10.1371/journal.pone.0225217 (PMC6892534; doi:10.1371/journal.pone.0225217)
Supplement: S1 Appendix — (DOCX) [file pone.0225217.s001.docx]

# S1 Appendix: Code used to simulate and analyse data

#get packages

library(ggplot2) ; library(nlme); library(dagitty)

#set seed

set.seed(42)

ctrl<-lmeControl(opt='optim', maxIter=200)

for (mainweight in c("mainweight0", "mainweight1", "mainweight2")){

sink(file=paste("simulate_in_parts", mainweight, ".txt", sep=""), type="output", split=T)

#set empty vectors

zscore0<-numeric(length=0) #high outcome z-score measure 0

zscore1<-numeric(length=0) #high outcome z-score measure 1

zscore2<-numeric(length=0) #high outcome z-score measure 2

bzscore0<-numeric(length=0) #low outcome z-score measure 0

bzscore1<-numeric(length=0) #low outcome z-score measure 1

bzscore2<-numeric(length=0) #low outcome z-score measure 2

mean0<-numeric()

mean1<-numeric()

mean2<-numeric()

mean0low<-numeric()

mean1low<-numeric()

mean2low<-numeric()

mean0high<-numeric()

mean1high<-numeric()

mean2high<-numeric()

cor0<-numeric(length=0)

cor1<-numeric(length=0)

cor2<-numeric(length=0)

reg0<-numeric() #univariable regression coefficients

reg1<-numeric()

reg2<-numeric()

mlmbout<-numeric() #mlm outcome coefficient

mlmbtime<-numeric() #mlm time coefficient

mlmbintr<-numeric() #mlm interaction term coefficient

mlmcons<-numeric() #mlm constant

mlm.rand.intr<-numeric() #mlm random intercept

mlm.resid<-numeric() #mlm residuals

mlm.rand.time<-numeric()

mlm.is.cor<-numeric()

mlm.phi<-numeric()

fit0ab<-numeric() #fitted values high outcome

fit1ab<-numeric()

fit2ab<-numeric()

fit0be<-numeric() #fitted values low outcome

fit1be<-numeric()

fit2be<-numeric()

sd0<-numeric()

sd1<-numeric()

sd2<-numeric()

meansbp<-numeric()

sdsbp<-numeric()

modint<-numeric() #exposure model parameters, intercept

modtime<-numeric() #time

modrint<-numeric() #random intercept

modrtime<-numeric() #random slope

modres<-numeric() #residuals

modiscor<-numeric()

logist.modslope<-numeric() #slope coefficient in model of outcome on slope of above model

logist.modcons<-numeric()

logist.modinterceptcoef<-numeric()#residual

logist.modslope.exp<-numeric() #slope coefficient in model of outcome on slope of above model

logist.modcons.exp<-numeric()

logist.modinterceptcoef.exp<-numeric()

#set number of observations and simulations

n<-1000

nsim<-1000

marks<-nsim/20

#vector to collect errors

error.counter<-numeric()

#loop through simulations

for (i in 1:nsim) {

#set up DAG with DAGITTY

if (mainweight=="mainweight0"){

g<-dagitty("dag{

U -> growth [beta=0.5];

U -> zs0 [beta=-0.8];

growth -> zs1 [beta=0.4];

growth -> zs2 [beta=0.4];

zs0 -> zs1 [beta=0.2];

zs1 -> zs2 [beta=0.2];

zs0 -> outcome [beta=0.7];

zs1 -> outcome [beta=0];

zs2 -> outcome [beta=0];

U [unobserved];

growth [unobserved]

}"

)

}

if (mainweight=="mainweight1"){

g<-dagitty("dag{

U -> growth [beta=0.5];

U -> zs0 [beta=-0.8];

growth -> zs1 [beta=0.4];

growth -> zs2 [beta=0.4];

zs0 -> zs1 [beta=0.2];

zs1 -> zs2 [beta=0.2];

zs0 -> outcome [beta=0];

zs1 -> outcome [beta=0.7];

zs2 -> outcome [beta=0];

U [unobserved];

growth [unobserved]

}"

)

}

if (mainweight=="mainweight2"){

g<-dagitty("dag{

U -> growth [beta=0.5];

U -> zs0 [beta=-0.8];

growth -> zs1 [beta=0.4];

growth -> zs2 [beta=0.4];

zs0 -> zs1 [beta=0.2];

zs1 -> zs2 [beta=0.2];

zs0 -> outcome [beta=0];

zs1 -> outcome [beta=0];

zs2 -> outcome [beta=0.7];

U [unobserved];

growth [unobserved]

}"

)

}

coordinates(g)<-

list(x=c(growth= 1,

zs0= 0,

zs1= 2,

zs2= 4,

outcome=5,

U= -1),

y=c(growth= 1,

zs0= 2,

zs1= 2,

zs2= 2,

outcome=3,

U= 1.5))

#plot DAG

png(filename=paste("DAG_", mainweight, ".png", sep=""), width=8, height=6, units="in", res=100)

plot(g)

dev.off()

#simulate data

data<-data.frame(simulateSEM(g, N=n))

data$var0<-data$zs0*2+4

data$var1<-data$zs1*2+8

data$var2<-data$zs2*2+12

data$outcome<-data$outcome+5.8

data$above<-c(rep(0, n))

data$above[which(data$outcome>=6.5)]<-1

#reshape data

data$id<-c(1:n)

data.long<-reshape(data, idvar="id", direction="long", timevar="time", sep="", varying=c("var0", "var1", "var2", "zs0", "zs1", "zs2"))

data.long$above<-factor(data.long$above)

data.long$timec<-data.long$time-1

#multilevel model with outcome as covariate

mlmod<-tryCatch({expr=lme(var~1 + above + timec + above*timec, random= ~ 1+timec|id, data=data.long, control=ctrl, cor=corAR1(form=~timec|id))},

error=function(e){return(NA)})

if(is.na(mlmod)==F){

data.long$fitted<-predict(mlmod, re.form=NA)

fit0ab<-c(fit0ab, mean(data.long$fitted[which(data.long$time==0&data.long$above==1)]))

fit1ab<-c(fit1ab, mean(data.long$fitted[which(data.long$time==1&data.long$above==1)]))

fit2ab<-c(fit2ab, mean(data.long$fitted[which(data.long$time==2&data.long$above==1)]))

fit0be<-c(fit0be, mean(data.long$fitted[which(data.long$time==0&data.long$above==0)]))

fit1be<-c(fit1be, mean(data.long$fitted[which(data.long$time==1&data.long$above==0)]))

fit2be<-c(fit2be, mean(data.long$fitted[which(data.long$time==2&data.long$above==0)]))

coefs<-apply(data.frame(coef(mlmod)), 2, mean)

mlmbout<-c(mlmbout, coefs[2])

mlmbtime<-c(mlmbtime, coefs[3])

mlmbintr<-c(mlmbintr, coefs[4])

mlmcons<-c(mlmcons, coefs[1])

mlm.rand.intr<-c(mlm.rand.intr, as.numeric(VarCorr(mlmod)[1,2]))

mlm.resid<-c(mlm.resid, as.numeric(VarCorr(mlmod)[3,2]))

mlm.rand.time<-c(mlm.rand.time, as.numeric(VarCorr(mlmod)[2,2]))

mlm.is.cor<-c(mlm.is.cor, as.numeric(VarCorr(mlmod)[2,3]))

mlm.phi<-c(mlm.phi, coef(mlmod$modelStruct$corStruct, unconstrained=F))

}else{

fit0ab<-c(fit0ab, NA)

fit1ab<-c(fit1ab, NA)

fit2ab<-c(fit2ab, NA)

fit0be<-c(fit0be, NA)

fit1be<-c(fit1be, NA)

fit2be<-c(fit2be, NA)

mlmbout<-c(mlmbout, NA)

mlmbtime<-c(mlmbtime, NA)

mlmbintr<-c(mlmbintr, NA)

mlmcons<-c(mlmcons, NA)

mlm.rand.intr<-c(mlm.rand.intr, NA)

mlm.resid<-c(mlm.resid, NA)

mlm.rand.time<-c(mlm.rand.time, NA)

error.counter<-c(error.counter, i+0.1)

}

#multilevel model in two-step process

mlmodgrp<-tryCatch({expr=lme(fixed=var~1 + timec, random= ~ 1+timec|id, data=data.long, control=ctrl, cor=corAR1(form=~timec|id))},

error=function(b){return(NA)})#random slope model for exposure in terms of time

if(is.na(mlmodgrp)==F){

modint<-c(modint, mlmodgrp$coefficients$fixed[1])

modtime<-c(modtime, mlmodgrp$coefficients$fixed[2])

modrint<-c(modrint, as.numeric(VarCorr(mlmodgrp)[1,2]))

modrtime<-c(modrtime, as.numeric(VarCorr(mlmodgrp)[2,2]))

modres<-c(modres, as.numeric(VarCorr(mlmodgrp)[3,2]))

modiscor<-c(modiscor, as.numeric(VarCorr(mlmodgrp)[2,3]))

data$slope<-mlmodgrp$coefficients$random$id[,"timec"]+mlmodgrp$coefficients$fixed[2]

data$slope10<-data$slope*10

data$intercept<-mlmodgrp$coefficients$random$id[,"(Intercept)"]+mlmodgrp$coefficients$fixed[1]

data$above<-factor(data$above)

mod<-tryCatch({espr=glm(above~slope10 + var0

, family=binomial(link="logit"), data=data)},

error=function(c){return(NA)})

if(is.na(mod)==F){#logistic model for outcome in terms of slope

logist.modslope<-c(logist.modslope, coefficients(mod)[2])

logist.modcons<-c(logist.modcons, coefficients(mod)[1])

logist.modinterceptcoef<-c(logist.modinterceptcoef, coefficients(mod)[3])

logist.modslope.exp<-c(logist.modslope.exp, exp(coefficients(mod)[2]))

logist.modcons.exp<-c(logist.modcons.exp, exp(coefficients(mod)[1]))

logist.modinterceptcoef.exp<-c(logist.modinterceptcoef.exp, exp(coefficients(mod)[3]))

}else{

logist.modslope<-c(logist.modslope, NA)

logist.modcons<-c(logist.modcons, NA)

logist.modslope.exp<-c(logist.modslope.exp, NA)

logist.modcons.exp<-c(logist.modcons.exp, NA)

error.counter=c(error.counter, i+0.3)

}

} else {

modint<-c(modint, NA)

modtime<-c(modtime, NA)

modrint<-c(modrint, NA)

modrtime<-c(modrtime, NA)

modres<-c(modres, NA) #logistic model for outcome in terms of slope

logist.modslope<-c(logist.modslope, NA)

logist.modcons<-c(logist.modcons, NA)

logist.modslope.exp<-c(logist.modslope.exp, NA)

logist.modcons.exp<-c(logist.modcons.exp, NA)

error.counter=c(error.counter, i+0.2)

}

#record z scores for high outcome group and variable outcome correlations

zscore0<-c(zscore0, mean(data$zs0[which(data$above==1)]))

zscore1<-c(zscore1, mean(data$zs1[which(data$above==1)]))

zscore2<-c(zscore2, mean(data$zs2[which(data$above==1)]))

bzscore0<-c(bzscore0, mean(data$zs0[which(data$above==0)]))

bzscore1<-c(bzscore1, mean(data$zs1[which(data$above==0)]))

bzscore2<-c(bzscore2, mean(data$zs2[which(data$above==0)]))

mean0high<-c(mean0high, mean(data$var0[which(data$above==1)]))

mean1high<-c(mean1high, mean(data$var1[which(data$above==1)]))

mean2high<-c(mean2high, mean(data$var2[which(data$above==1)]))

mean0low<-c(mean0low, mean(data$var0[which(data$above!=1)]))

mean1low<-c(mean1low, mean(data$var1[which(data$above!=1)]))

mean2low<-c(mean2low, mean(data$var2[which(data$above!=1)]))

mean0<-c(mean0, mean(data$var0))

mean1<-c(mean1, mean(data$var1))

mean2<-c(mean2, mean(data$var2))

cor0<-c(cor0, cor(data$var0, data$outcome))

cor1<-c(cor1, cor(data$var1, data$outcome))

cor2<-c(cor2, cor(data$var2, data$outcome))

sd0<-c(sd0, sd(data$var0))

sd1<-c(sd1, sd(data$var1))

sd2<-c(sd2, sd(data$var2))

meansbp<-c(meansbp, mean(data$outcome))

sdsbp<-c(sdsbp, sd(data$outcome))

}

#collect results

results<-data.frame(cbind(zscore0, zscore1, zscore2, bzscore0, bzscore1, bzscore2, mean0, mean1, mean2, mean0low, mean1low, mean2low,

mean0high, mean1high, mean2high, cor0, cor1, cor2, reg0, reg1, reg2, mlmbout, mlmbtime, mlmbintr, mlmcons,

mlm.rand.intr, mlm.resid,mlm.phi, fit0ab, fit1ab, fit2ab, fit0be, fit1be, fit2be, logist.modslope,logist.modcons, logist.modinterceptcoef,

modint, modtime, modrint, modrtime, modres, sd0, sd1, sd2 , meansbp, sdsbp, mlm.rand.time, mlm.is.cor, modiscor, logist.modslope.exp, logist.modcons.exp, logist.modinterceptcoef.exp

))

write.csv(results, file=paste("resultsdata_", mainweight, ".csv", sep=""))

write.csv(error.counter, file=paste("error.counter", mainweight, ".csv", sep=""))

sink(file=NULL)

}

#analyse each set of results

for (mainweight in c("mainweight0", "mainweight1", "mainweight2")){

sink(file=paste("analyse_simulate_in_parts", mainweight, ".txt", sep=""), type="output", split=T)

data<-read.csv(file=paste("resultsdata_", mainweight, ".csv", sep=""))

#collect errors and remove results from these simulations

errors<-read.csv(file=paste("error.counter", mainweight, ".csv", sep=""))

errors<-round(errors[,2], 0)

data[errors,]<-NA

#get means and quantiles of simulation results

isna<-apply(data,1,function(x) sum(is.na(x)))

allmeans<-apply(data[which(isna==0),], 2, mean, na.rm=T)

allquantiles<-apply(data[which(isna==0),], 2, quantile, probs=c(0.025, 0.975, 0.5), na.rm=T)

#record zscores in groups

zscores<-c(allmeans["zscore0"], allmeans["zscore1"], allmeans["zscore2"])

zscores95<-c(allquantiles[2,"zscore0"],allquantiles[2,"zscore1"],allquantiles[2,"zscore2"])

zscores5<-c(allquantiles[1,"zscore0"],allquantiles[1,"zscore1"],allquantiles[1,"zscore2"])

bzscores95<-c(allquantiles[2,"bzscore0"],allquantiles[2,"bzscore1"],allquantiles[2,"bzscore2"])

bzscores5<-c(allquantiles[1,"bzscore0"],allquantiles[1,"bzscore1"],allquantiles[1,"bzscore2"])

bzscores<-c(allmeans["bzscore0"], allmeans["bzscore1"], allmeans["bzscore2"])

#record means in groups

means<-c(allmeans["mean0"], allmeans["mean1"], allmeans["mean2"])

highmeans<-c(allmeans["mean0high"], allmeans["mean1high"], allmeans["mean2high"])

lowmeans<-c(allmeans["mean0low"], allmeans["mean1low"], allmeans["mean2low"])

#record mlm fitted values for high and low outcome groups

fitabove<-c(allmeans["fit0ab"], allmeans["fit1ab"], allmeans["fit2ab"])

fitbelow<-c(allmeans["fit0be"], allmeans["fit1be"], allmeans["fit2be"])

fitabove5<-c(allquantiles[1, "fit0ab"], allquantiles[1, "fit1ab"], allquantiles[1, "fit2ab"])

fitabove95<-c(allquantiles[2, "fit0ab"], allquantiles[2, "fit1ab"], allquantiles[2, "fit2ab"])

fitbelow5<-c(allquantiles[1, "fit0be"], allquantiles[1, "fit1be"], allquantiles[1, "fit2be"])

fitbelow95<-c(allquantiles[2, "fit0be"], allquantiles[2, "fit1be"], allquantiles[2, "fit2be"])

time<-c(0,1,2)

#plot z-scores

dat<-data.frame(cbind(c(zscores, bzscores), c(zscores5, bzscores5), c(zscores95, bzscores95), c(time, time), c(1,1,1,0,0,0), c(rep(c(-3,-3,3),2))))

colnames(dat)<-c("mean.zscore", "lower", "upper", "time", "Outcome", "hjust")

dat$Outcome<-factor(dat$Outcome)

png(filename=paste("mean_zscores_simulate_in_parts", mainweight, ".png", sep=""), width=8, height=6, units="in", res=100)

print(ggplot(data=dat, aes(x=time, y=mean.zscore, group=Outcome))+

theme_bw()+

geom_errorbar(data=dat, aes(x=time, ymin=lower, ymax=upper, colour=Outcome, width=0.05), show.legend=F)+

geom_point(size=1.5, aes(colour=Outcome))+

geom_line(size=1, aes(linetype=Outcome, colour=Outcome))+

scale_linetype_manual(values=c("dashed", "dotted"), breaks=c(1,0),name="Diabetes")+

scale_colour_discrete(breaks=c(1,0), name="Diabetes")+

geom_hline(yintercept=0, colour="grey16")+

labs(x="Age (years)", y="Mean weight z-score"))

dev.off()

#plot fitted values from mlm with confidence intervals

dat<-data.frame(cbind(c(fitabove, fitbelow), c(fitabove5, fitbelow5), c(fitabove95, fitbelow95), c(time, time), c(1,1,1,0,0,0)))

names(dat)<-c("MLM_preds_fix", "lower", "upper", "time", "grp")

dat2<-data.frame(cbind(c( highmeans, lowmeans), c( time, time), c(4,4,4,5,5,5)))

colnames(dat2)<-c("means", "time", "grp")

png(filename=paste("MLM_fixed_preds_simulate_in_parts", mainweight, ".png", sep=""), width=8, height=6, units="in", res=100)

print(ggplot() +

theme_bw()+

geom_line(data=dat,size=1, colour="grey16", aes(x=time, y=MLM_preds_fix, group=factor(grp), linetype=factor(grp)))+

geom_ribbon(data=dat, aes(x=time, ymin=lower, ymax=upper, group=grp), alpha=0.2)+

geom_point(data=dat2, aes(x=time, y=means, colour=factor(grp), shape=factor(grp)), size=2)+

scale_linetype_manual(values=c("dashed", "dotted"), name="MLM fitted values", breaks=c(1,0), labels=c("Diabetes", "No diabetes"))+

scale_colour_discrete(name="Mean values", breaks=c(3,4,5), labels=c("All", "Diabetes", "No diabetes"))+

scale_shape_discrete(name="Mean values", breaks=c(3,4,5), labels=c("All", "Diabetes", "No diabetes"))+

scale_y_continuous(minor_breaks=seq(0, 95, 0.5))+

labs(x="Age (years)", y="Weight(kg)"))

dev.off()

#generate tables as per paper (table 1 based on inputs for simulation)

tab2<-data.frame(cbind(c(paste(round(allmeans["mean0"],3), "(", round(allquantiles[1, "mean0"],3), ", ", round(allquantiles[2, "mean0"],3), ")", sep=""), paste(round(allmeans["sd0"],3), "(", round(allquantiles[1, "sd0"],3), ", ", round(allquantiles[2, "sd0"],3), ")", sep=""), paste(round(allmeans["cor0"],3), "(", round(allquantiles[1, "cor0"],3), ", ", round(allquantiles[2, "cor0"],3), ")", sep="")),

c(paste(round(allmeans["mean1"],3), "(", round(allquantiles[1, "mean1"],3), ", ", round(allquantiles[2, "mean1"],3), ")", sep=""), paste(round(allmeans["sd1"],3), "(", round(allquantiles[1, "sd1"],3), ", ", round(allquantiles[2, "sd1"],3), ")", sep=""), paste(round(allmeans["cor1"],3), "(", round(allquantiles[1, "cor1"],3), ", ", round(allquantiles[2, "cor1"],3), ")", sep="")),

c(paste(round(allmeans["mean2"],3), "(", round(allquantiles[1, "mean2"],3), ", ", round(allquantiles[2, "mean2"],3), ")", sep=""), paste(round(allmeans["sd2"],3), "(", round(allquantiles[1, "sd2"],3), ", ", round(allquantiles[2, "sd2"],3), ")", sep=""), paste(round(allmeans["cor2"],3), "(", round(allquantiles[1, "cor2"],3), ", ", round(allquantiles[2, "cor2"],3), ")", sep="")),

c(paste(round(allmeans["meansbp"],3), "(", round(allquantiles[1, "meansbp"],3), ", ", round(allquantiles[2, "meansbp"],3), ")", sep=""), paste(round(allmeans["sdsbp"],3), "(", round(allquantiles[1, "sdsbp"],3), ", ", round(allquantiles[2, "sdsbp"],3), ")", sep=""), "1")))

colnames(tab2)<-c("Weight0", "Weight1", "Weight2", "HBA1C")

rownames(tab2)<-c("Mean (95% CI)", "SD (95% CI)", "Mean correlation with HBA1C (95% CI)")

tab3<-data.frame(cbind(c(round(allquantiles[3,"mlmbout"],3), round(allquantiles[3,"mlmbtime"],3), round(allquantiles[3,"mlmbintr"],3), round(allquantiles[3,"mlmcons"],3), round(allquantiles[3,"mlm.rand.intr"],3), round(allquantiles[3,"mlm.rand.time"],3), round(allquantiles[3,"mlm.resid"],3), round(allquantiles[3,"mlm.is.cor"],3), round(allquantiles[3,"mlm.phi"],3)),

c(paste(round(allquantiles[1,"mlmbout"],3), ", ", round(allquantiles[2,"mlmbout"],3), sep=""),

paste(round(allquantiles[1,"mlmbtime"],3), ", ", round(allquantiles[2,"mlmbtime"],3), sep=""),

paste(round(allquantiles[1,"mlmbintr"],3), ", ", round(allquantiles[2,"mlmbintr"],3), sep=""),

paste(round(allquantiles[1,"mlmcons"],3), ", ", round(allquantiles[2,"mlmcons"],3), sep=""),

paste(round(allquantiles[1,"mlm.rand.intr"],3), ", ", round(allquantiles[2,"mlm.rand.intr"],3), sep=""),

paste(round(allquantiles[1,"mlm.rand.time"],3), ", ", round(allquantiles[2,"mlm.rand.time"],3), sep=""),

paste(round(allquantiles[1,"mlm.resid"],3), ", ", round(allquantiles[2,"mlm.resid"],3), sep=""),

paste(round(allquantiles[1,"mlm.is.cor"],3), ", ", round(allquantiles[2,"mlm.is.cor"],3), sep=""),

paste(round(allquantiles[1, "mlm.phi"],3), ",", round(allquantiles[2, "mlm.phi"],3), sep=""))))

colnames(tab3)<-c("Median Parameter estimate", "95% CI")

rownames(tab3)<-c("Diabetes", "Age", "Diabetes*Age", "Constant", "Contant Variance", "Age Variance", "Residual Variance", "Constant-Age Covariance", "Autocorrelation parameter")

tab4<-data.frame(cbind(c(round(allquantiles[3,"logist.modslope.exp"],3), round(allquantiles[3,"logist.modinterceptcoef.exp"]),

round(allquantiles[3,"logist.modcons.exp"],3)),

c(paste(round(allquantiles[1,"logist.modslope.exp"],3), ", ", round(allquantiles[2,"logist.modslope.exp"],3), sep=""),

paste(round(allquantiles[1,"logist.modinterceptcoef.exp"],3), ", ", round(allquantiles[2,"logist.modinterceptcoef.exp"],3), sep=""),

paste(round(allquantiles[1,"logist.modcons.exp"],3), ", ", round(allquantiles[2,"logist.modcons.exp"],3), sep=""))))

colnames(tab4)<-c("Median Odds ratio", "95% CI")

rownames(tab4)<-c("Growth rate", "Weight0", "Constant")

write.csv(tab2, file=paste("tab2", mainweight, ".csv", sep=""))

write.csv(tab3, file=paste("tab3", mainweight, ".csv", sep=""))

write.csv(tab4, file=paste("tab4", mainweight, ".csv", sep=""))

write.csv(allmeans, file=paste("allmeans", mainweight, ".csv", sep=""))

write.csv(allquantiles, file=paste("allquantiles", mainweight, ".csv", sep=""))

print(allmeans)

print(allquantiles)

print(paste("N errors:", length(errors), sep=" "))

eval(parse(text=paste("logmod", mainweight, "<-data$logist.modslope.exp[which(isna==0)]", sep="")))

eval(parse(text=paste("mlmbintr", mainweight, "<-data$mlmbintr[which(isna==0)]", sep="")))

sink(file=NULL)

}
