# Supplementary material for: Analysing trajectories of a longitudinal exposure: A causal perspective on common methods in lifecourse research
Source: PLoS One. 2019 Dec 4;14(12):e0225217. doi: 10.1371/journal.pone.0225217 (PMC6892534; doi:10.1371/journal.pone.0225217)
Supplement: Attachment — Submitted filename: Response to reviewer comments.docx [file pone.0225217.s002.docx]

# Responses to reviewer comments

**PONE-D-19-21843: Analysing trajectories of a longitudinal exposure: a causal perspective on common methods in lifecourse research**

Thank you for the opportunity to revise our paper. We are grateful to editors and reviewers for the thoughtful comments and instructions that have helped us to further improve the paper. We have responded to individual comments and instructions below.

## Additional requirements

1. Please ensure that your manuscript meets PLOS ONE's style requirements, including those for file naming. The PLOS ONE style templates can be found at <http://www.journals.plos.org/plosone/s/file?id=wjVg/PLOSOne_formatting_sample_main_body.pdf> and <http://www.journals.plos.org/plosone/s/file?id=ba62/PLOSOne_formatting_sample_title_authors_affiliations.pdf>

**Author response:** Thank you for providing these formatting samples.

**Author changes:** Formatting has been amended throughout the paper. The manuscript file has been named “Manuscript.docx”.

2. In your Methods section, it is reported that "Simulation code is available as supplementary material"; please correct this statement, as we note that the code is not provided as a supplementary file, but is instead deposited in GitHub.

**Author response:** We appreciate identification of this error. We have chosen to include code as supplementary material as GitHub hosting has the potential to not be permanent.

**Author change:** Changed “as supplementary material.” to “in the S1 appendix.” in Methods [p5]; Added “Supporting information¶ S1 Appendix: Code used to simulate and analyse data.” at end of manuscript [p19]. Supplementary material has been submitted in the file “S1_Appendix.docx”.

## Reviewer #1 Questions

1. Is the manuscript technically sound, and do the data support the conclusions?

Reviewer #1: Yes

2. Has the statistical analysis been performed appropriately and rigorously?

Reviewer #1: Yes

3. Have the authors made all data underlying the findings in their manuscript fully available?

Reviewer #1: No

**Author response:** The data underlying findings in this manuscript was simulated using code made available on GitHub which we appreciate was not correctly indicated in the manuscript. Following on from this, we decided to include code as an appendix as GitHub hosting may not be permanent.

**Author change:** Changed “as supplementary material.” to “in the S1 appendix.” in Methods [p5]; Added “Supporting Information Captions¶ S1 Appendix: Code used to simulate and analyse data.” at end of manuscript [p19]. Supplementary material has been submitted in the file “S1_Appendix.docx”.

4. Is the manuscript presented in an intelligible fashion and written in standard English?

Reviewer #1: Yes

## Reviewer #1 Comments to the Author

This is an interesting paper, addressing a very important methodological issue in lifecourse epidemiology. I have some suggestions to improve the accessibility of this manuscript:

1. To make the comparison across different models/methodologies more explicit, I think it would be useful to spell out the model specifications in equations. Although the directed acyclic diagrams are useful for visualizing the causal relations between variables, equations help readers with the understanding of the statistical models used to estimate the causal relations.

**Author response:** Thank you for this suggestion, we agree that this will be a useful addition to the paper to clarify the models used. We note that no equations have been provided for the Z-score plots as these do not use models.

**Author change:** Equation 1 added following the changed sentence: “The multilevel model (outcome as covariate) analysis involved fitting multi-level models of weight over time, with covariates for age, diabetes status, and an age-diabetes interaction term, defined by the following equations (where *i* indexes observations and *j* individuals):“ in Methods [p6]; Equation 2 added following the altered sentence: “The multilevel model (two-step) approach involved fitting two models, defined by the following equations (where *i* indexes observations and *j* individuals):” in Methods [p6-7]. References to Equation 2.1 and 2.2 added in Methods [p7]: “The first (Equation 2.1) was a multilevel…” and “In the second step (Equation 2.2), a logistic…”

2. Since DAGs are used to show the causal structure in the simulated data, I think it would be equally useful to use DAGs to show the implied causal structures within the statistical models (e.g. multilevel models etc). SO readers could compare the DAGs to apprehend why some models are more "correct" than others in different scenarios.

**Author response:** Thanks for this useful suggestion. While we agree that visual representations of models are often useful for explaining them, in this case, we have chosen not to include DAGs to represent the implied causal structures of the models for the following reasons:

1. **A single model specification could correctly estimate a causal effect for a multitude of different DAGs.** That is, a DAG (given a chosen exposure and outcome) implies one (or several) appropriate models, but a given model may also be appropriate for many DAGs. For example, all of the DAGs below imply the model D~A to find the total causal effect of A on D, but the model implies any of these DAGs (and many more with these four variables).

A

B

D

C

A

B

D

C

A

B

D

C

A

B

D

C

We thought to include many implied DAGs for each model could potentially lead to greater confusion than clarity and would certainly warrant a more detailed explanation that might only detract from the main message of the paper.

2. **Some features of the models are not commonly understood as a feature of DAGs.** In particular, this relates to the multilevel model with an outcome as covariate which contains an interaction term, indicating “effect modification” between the time and diabetes variables. While effect modification in DAGs has been discussed (<https://www.ncbi.nlm.nih.gov/pubmed/17700242>) it is not commonly implemented and so would not be a familiar component of a DAG to most readers.

In the presence of the newly included model equations to clarify their specification (as per comment 1) we felt that the inclusion of implied DAGs may create more confusion than the clarity it would seek to bring.

3. Although the causal relations between the variables in the simulated data were shown in DAGs, I feel it would useful to provide the R codes in the appendix; so readers could examine the details of the simulations.

**Author response:** Thank you for highlighting this issue – we have made appropriate changes to include the code as supplementary material as discussed in our response to Reviewer #1 Questions #3.

## Reviewer #2 Questions

1. Is the manuscript technically sound, and do the data support the conclusions?

Reviewer #2: Yes

2. Has the statistical analysis been performed appropriately and rigorously?

Reviewer #2: Yes

3. Have the authors made all data underlying the findings in their manuscript fully available?

Reviewer #2: Yes

4. Is the manuscript presented in an intelligible fashion and written in standard English?

Reviewer #2: Yes

## Reviewer #2 Comments to the Author

This is a clear, nicely written paper on an important topic. My comments are below:

Major comment:

1. The methods that condition on the outcome didn't perform as badly as I expected. My understanding is that this is because of the restriction to settings with no time-varying confounder-treatment feedback as stated in the discussion. I think it would be helpful to introduce this restriction / assumption earlier in the paper -- perhaps in the introduction -- so that the results don't get over-generalized.

**Author response:** Thank you for this useful comment, we agree that it is important to introduce this earlier in the paper for the reasons stated and we have made changes to do so.

**Author changes:** In the Introduction section [p4] the sentence “Approaches are suggested to avoid these biases” has been changed to “Such methods are compared to an alternative approach that avoids these biases. This method, however, is not suitable for all situations; other methods, such as g-methods, would be necessary in the presence of time-varying confounding, which is not examined in this paper.”

Minor comment:

1. The simulated data creates a scenario in which no one is lost to follow-up or experiences a competing event. While this is a reasonable simplification to demonstrate the problems with the outcome-control methods, some readers may miss that there are further potential dangers associated with LTFU or competing events. A short review of these issues in the discussion would make the paper a bit more complete.

**Author response:** This is another important restriction that should be highlighted and we are grateful for pointing it out.

**Author changes:** We have added the following paragraph to the discussion section [p15]: “It is important to note that, for illustrative purposes, this paper presents a simplified scenario in which there are no competing events or loss to follow up, both of which would be present in reality. Any differential loss to follow up or occurrence of competing events would (further) bias the results from all three methods examined in this paper [20].” Reference number 20 has been added to the reference list [p19].
